# High‐resolution temporal dynamic transcriptome landscape reveals a *GhCAL*‐mediated flowering regulatory pathway in cotton (*Gossypium hirsutum* L.)

**DOI:** 10.1111/pbi.13449

**Published:** 2020-08-03

**Authors:** Shuaishuai Cheng, Pengyun Chen, Zhengzheng Su, Liang Ma, Pengbo Hao, Jingjing Zhang, Qiang Ma, Guoyuan Liu, Ji Liu, Hantao Wang, Hengling Wei, Shuxun Yu

**Affiliations:** ^1^ College of Agronomy Northwest A&F University Yangling China; ^2^ State Key Laboratory of Cotton Biology Key Laboratory of Cotton Genetic Improvement Cotton Institute of the Chinese Academy of Agricultural Sciences Ministry of Agriculture Anyang China

**Keywords:** *Gossypium hirsutum*, early maturity, flower bud differentiation, transcriptome, *GhCAL*

## Abstract

The transition from vegetative to reproductive growth is very important for early maturity in cotton. However, the genetic control of this highly dynamic and complex developmental process remains unclear. A high‐resolution tissue‐ and stage‐specific transcriptome profile was generated from six developmental stages using 72 samples of two early‐maturing and two late‐maturing cotton varieties. The results of histological analysis of paraffin sections showed that flower bud differentiation occurred at the third true leaf stage (3TLS) in early‐maturing varieties, but at the fifth true leaf stage (5TLS) in late‐maturing varieties. Using pairwise comparison and weighted gene co‐expression network analysis, 5312 differentially expressed genes were obtained, which were divided into 10 gene co‐expression modules. In the MElightcyan module, 46 candidate genes regulating cotton flower bud differentiation were identified and expressed at the flower bud differentiation stage. A novel key regulatory gene related to flower bud differentiation, *GhCAL*, was identified in the MElightcyan module. *Anti‐GhCAL* transgenic cotton plants exhibited late flower bud differentiation and flowering time. GhCAL formed heterodimers with GhAP1‐A04/GhAGL6‐D09 and regulated the expression of *GhAP1‐A04* and *GhAGL6‐D09*. *GhAP1‐A04*‐ and *GhAGL6‐D09*‐silenced plants also showed significant late flowering. Finally, we propose a new flowering regulatory pathway mediated by *GhCAL*. This study elucidated the molecular mechanism of cotton flowering regulation and provides good genetic resources for cotton early‐maturing breeding.

## Introduction

Upland cotton (*Gossypium hirsutum* L.) is the most important fibre crop in the world, which is widely cultivated in short days; however, its sensitivity to day length is lost in the process of domestication (Hao *et al*., 2008). Due to the conflict between food crops and upland cotton cultivation in cultivated land use, early maturity has become a key feature of China's short‐season cotton breeding programme (Song *et al*., 2012). Xinjiang is one of the largest ecological regions of early‐maturing cotton in China. There are many weather disasters in spring in the cotton areas of northern Xinjiang, and a certain area is replanted every year due to these disasters, resulting in late sowing dates and high risk of cotton planting, so there is an urgent need for early‐maturing cotton varieties in production (Li *et al*., 2002a). Flower bud differentiation is an important character affecting the early maturity of short‐season cotton varieties, and it is the basis of cotton development at the budding, flowering and boll‐setting stages (Fang *et al*., 2018). Flower bud differentiation directly affects flowering time (He, 2009; Moon *et al*., 2005). The top of the flower bud is relatively flat, cylindrical and larger in volume (Ren *et al*., 2000). Flower bud differentiation serves as an indicator of the transition of plants from vegetative to reproductive growth. Upon entering reproductive growth, lateral buds become flower buds, which develop into fruit branches. The differentiation of fruit branches determines the amount of flowering, sexual ability and cotton yield (Shen et al., 1989).

The transition from vegetative to reproductive growth is the most important event in the growth and development of higher plants. It is regulated by external environmental factors and internal genes (Mouradov *et al*., 2002). In the past few years, several transcriptome mapping studies have been conducted to identify flowering‐related genes (Fan *et al*., 2018; Jian *et al*., 2019; Shah *et al*., 2018; Song *et al*., 2017). The analysis of *Arabidopsis* bud meristem transcriptome during flowering transition has identified different regulation patterns and 202 candidate genes (Torti *et al*., 2012b). Global transcriptome analysis of rice has identified 357 differentially expressed genes in the early stage of panicle development from phase transition to floral organ differentiation (Furutani *et al*., 2006). However, most of the verified key genes for controlling phase transition were initially identified in model plants such as *A. thaliana* and rice (Blumel *et al*., 2015; Shrestha *et al*., 2014). To date, more than 300 functional genes are known to control flowering time of *A. thaliana* such as *FT* (Niwa *et al*., 2013), *SOC1* (Kimura *et al*., 2015) and *AGL24* (Torti and Fornara, 2012a). In maize, *DLF1* is a key gene necessary for inflorescence transition (Muszynski *et al*., 2006). *bHLH* plays an important role in flowering, fruit ripening and development of tomato (Waseem *et al*., 2019). Currently, among the verified flowering gene families, the MADS‐box gene family is one of the most important families because its members are widely conserved in angiosperm species and play a key role in reproductive development (Gramzow and Theissen, 2010; Ng and Yanofsky, 2000). *CsMADS02* is a potential regulator of flowering time and leaf morphology (Zhou *et al*., 2019). Overexpression of *AtCAL* in *A. thaliana* can lead to early flowering (Li *et al*., 2000). The *AtAP1* gene is not only a floral meristem characteristic gene, but also a floral organ morphological characteristic gene (Irish, 1990; Mandel *et al*., 1992). *AtAGL6* controls early flowering by limiting the expression of flowering inhibitors *FLC* and *MAF* (Koo *et al*., 2010).

Because of the perennial, uncertain growth and colocation of its wild ancestors, cotton has a complex growth pattern (Chen *et al*., 2015; McGarry *et al*., 2016). To explain the genetic basis of traits related to early maturity in cotton, numerous genetic linkage maps have been constructed (Fan *et al*., 2006; Guo *et al*., 2009; Guo *et al*., 2008; Lacape *et al*., 2013; Li *et al*., 2012; Li *et al*., 2013; Li *et al*., 2014; Liu *et al*., 2014). The results showed that early maturity was a complex quantitative trait that consisted of growth period (including sowing date, budding time, flowering time and boll‐opening time), pre‐frost yield (YPBF), node position of the first fruit branch (NFFB) and height of the node of the first fruit branch (HNFFB) (Li *et al*., 2013). Because of this complexity, no flowering‐related genes were isolated from cotton by map‐based cloning. So far, only a small number of genes have been cloned by reverse genetics and preliminarily verified that they may control flowering in cotton such as *GhFPF1* (Wang *et al*., 2014), *GhSOC1* (Zhang *et al*., 2016), *GhCEN‐DT* (Liu *et al*., 2018) and *GhAAI66* (Qanmber *et al*., 2019). Flower bud differentiation is an important character influencing the early maturity of cotton varieties. Research on cotton flower bud differentiation is limited to its morphology (Ren *et al*., 2000), whereas the key genes and regulatory mechanisms controlling cotton flower bud differentiation have not been reported to date.

Here, to study the molecular mechanism of cotton early maturity regulation network, we selected two early‐maturing and two late‐maturing varieties, collected shoot apex samples before and after flower bud differentiation and analysed their transcript dynamics. Finally, we found a key regulatory gene that regulated the transition from vegetative growth to reproductive growth in cotton, and verified its function by transgenic plants. This study will benefit the development of early‐maturing varieties in cotton breeding.

## Results

### Morphological development of cotton shoot apex

**Figure 1:**
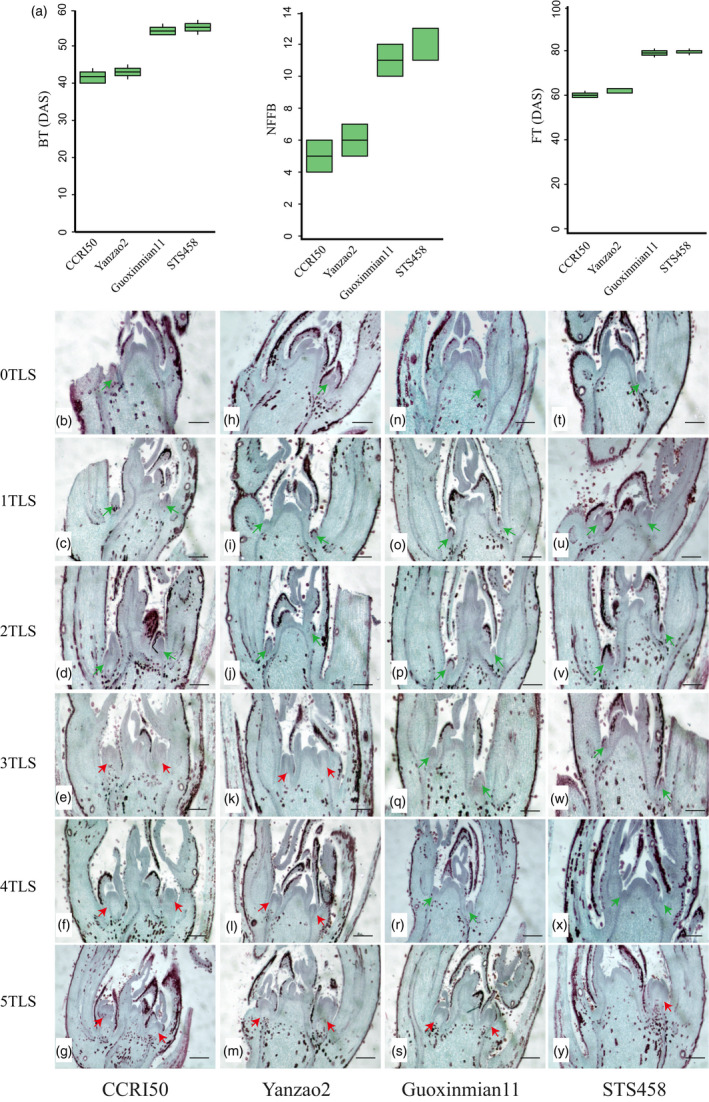
Comparison of two early‐maturing and two late‐maturing varieties. (a) Comparison of characters related to early maturity. BT, budding time; DAS, days after sowing; FT, flowering time; NFFB, node of the first fruiting branch. (b–y) Paraffin sections of cotton shoot apexes. 0TLS, 1TLS, 2TLS, 3TLS, 4TLS and 5TLS indicate the cotyledon, first, second, third, fourth and fifth true leaf stages, respectively. The green arrow represents the vegetative buds. The red arrow indicates the flower buds. Scale bars, 135 μm.

In this study, two early‐maturing *G. hirsutum* cultivars, CCRI50 and Yanzao2, and two late‐maturing cultivars, Guoxinmian11 and STS458, were selected for investigation. Compared with the late‐maturing varieties, budding time in the early‐maturing varieties was 10 days earlier, flowering time was 19 days earlier, and the whole growth period was 33 days shorter (Figure 1a).

After transferring to reproductive growth, the lateral buds became flower buds, which developed into fruiting branches. Histological analysis of paraffin sections showed that the axillary buds of the four varieties were conical at the cotyledon stage (0TLS), the first true leaf stage (1TLS) and the second true leaf stage (2TLS) (Figure 1b–d,h–j,n–p,t–v). In Yanzao2 and CCRI50, the flower buds appeared at the third true leaf stage (3TLS), and their tips were flat, large and cylindrical (Figure 1e–g,k–m). However, in Guoxinmian11 and STS458, at 3TLS and 4TLS, the axillary buds were always conical (Figure 1q–r,w–x), and the flower buds did not appear until 5TLS (Figure 1s,y). The above results showed that CCRI50 and Yanzao2 underwent earlier flower bud differentiation than Guoxinmian11 and STS458.

### Transcriptome profile of 72 RNA libraries from cotton plants at different developmental stages

**Figure 2:**
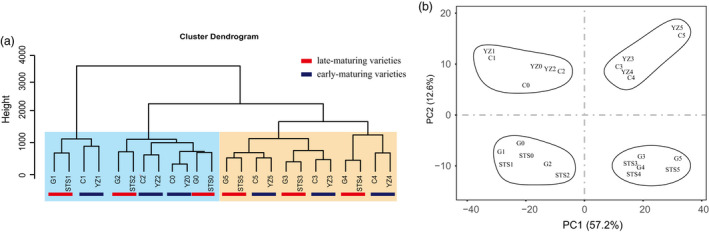
Transcriptome relationships among six developmental stages of early‐ and late‐maturing varieties. (a) The clustering tree diagram shows different clustering groups. (b) Principal component analysis of genes identified in all samples.

RNA‐sequencing (RNA‐seq) data were generated from 72 samples of four different varieties at six different developmental stages with three biological replicates. All the samples were named as the abbreviations of variety names and periods, that is the abbreviations of CCRI50, Yanzao2, Guoxinmian11 and STS458 were C, YZ, G and STS, respectively, and the abbreviations from cotyledon to fifth true leaf stages were 0, 1, 2, 3, 4 and 5. A total of three billion high‐quality clean reads were generated. The values of Q20 (~92%) and Q30 (~89%) indicated that the quality of the sequencing data was sufficient to support further transcriptome analysis. On average, about 85% of the reads were uniquely mapped (Table S1). The comparison of expression values among the three biological replicas was highly correlated (Figure S1). Therefore, the average FPKM values of the three replicates were calculated as the expression level of genes in each sample. To reduce the effects of transcriptional noise, genes with FPKM <0.5 were considered not expressed (Kang *et al*., 2013). A total of 49 000 genes were found to be expressed in at least one sample. To understand the transcriptional dynamics of the development of early‐ and late‐maturing cotton varieties, hierarchical clustering (Figure 2a) and principal component analysis (PCA) of all samples were performed (Figure 2b). The results showed that these high‐density time series transcripts could be initially divided into two categories according to the developmental stages, and then, in each category, early‐ and late‐maturing varieties were distinguished.

### Weighted gene co‐expression network analysis (WGCNA)

**Figure 3:**
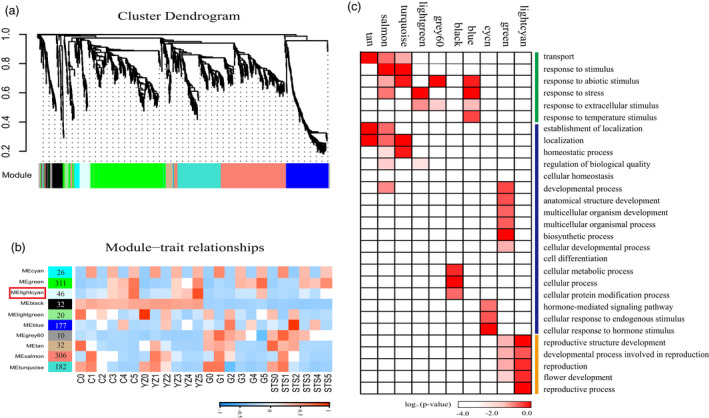
WGCNA of differentially expressed genes. (a) Hierarchical cluster tree showing co‐expression modules identified by WGCNA. Each leaf in the tree is one gene. The major tree branches constitute 10 modules labelled by different colours. (b) Module–sample association. Each row corresponds to a module. The name of modules is indicated on the left. Each column corresponds to a specific sample. The colour of each cell at the row–column intersection indicates the correlation coefficient between the module and sample. A high degree of correlation between a specific module and sample is indicated by red. (c) GO functional categories enriched by different co‐expression modules. Only significant categories (FDR < 0.05) are displayed.

An alternative analysis tool, WGCNA (Langfelder and Horvath, 2008), was adopted. Modules were defined as highly connected gene clusters; genes in the same cluster had a high correlation coefficient with each other. After screening for differentially expressed genes by FPKM (see Methods), 5312 genes were used for WGCNA, resulting in 10 different modules (marked with different colours) (Figure 3a,b and Table S2). Almost 80% of genes were clustered in development stage‐specific modules (MEcyan, Megreen, MElightgreen, MEtan, MEsalmon and MEturquoise) (Figure 3b). This was consistent with early hierarchical clustering (Figure 2a) and PCA (Figure 2b), and gene expression in the species was highly conserved, followed by the difference between the early‐ and late‐maturing varieties. The dynamic and stage‐ or variety‐specific expression patterns of these genes probably reflected the key functions they played. A total of 540 genes (including 33 transcription factors) were aggregated in the MElightgreen module (stages 0TLS–2TLS), the MEtan module (stages 0TLS and 2TLS), the MEsalmon module (stages 1TLS–2TLS) and the MEturquoise module (stages 0TLS–1TLS). According to the results of histological analysis of paraffin sections (Figure 1), there was no flower bud differentiation at 0TLS, 1TLS and 2TLS, which comprised the vegetative growth period. The results of GO analysis of this cluster showed that the GO terms were ‘transport’, ‘response to stimulus’, ‘response to abiotic stimulus’, ‘response to stress’, ‘response to extracellular stimulus’, ‘response to temperature stimulus’ (Figure 3c and Table S3). The genes in these modules may play an important role in cotton seedling responses to environmental changes to ensure their early normal growth and morphogenesis. The module Megreen contained a total of 311 genes, which were gradually up‐regulated with development among the four varieties. The reproductive growth‐related GO terms such as ‘flower development’, ‘reproductive structure development’, ‘developmental process involved in reproduction’ and ‘reproduction’ were enriched (Figure 3c). In addition, GO terms related to growth and development such as ‘developmental process’, ‘anatomical structure development’ and ‘multicellular organism development’ were also enriched. The genes in module MElightcyan were expressed at 3TLS, and their expressions in two early‐maturing varieties were always higher than that in two late‐maturing varieties (Figure 3b). GO enrichment analysis showed that these 46 genes enriched GO terms such as ‘flower development’, ‘developmental process involved in reproduction’, ‘reproductive structure development’ and ‘reproductive process’, which were related to reproductive growth (Figure 3c). Thus, it could be seen that the genes in this module played a vital role in transition of cotton from vegetative to reproductive growth and were worthy of being further analysed.

### Identification of genes involved in transition from vegetative to reproductive growth in cotton

**Figure 4:**
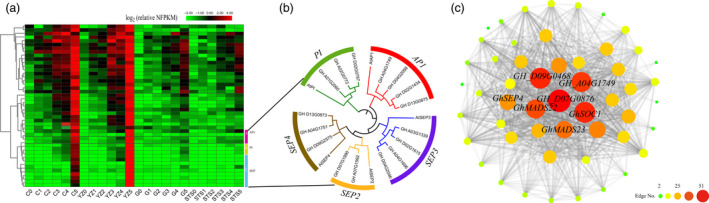
Analysis of module MElightcyan. (a) The heat map shows the relative NFPKM of genes from module MElightcyan. (b) Neighbour‐joining phylogenetic tree using 16 ABCDE homologous genes in module MElightcyan and *Arabidopsis thaliana* genes *AtAP1*, *AtPI*, *AtSEP2*, *AtSEP3* and *AtSEP4*. (c) The correlation network of module MElightcyan. A gene network is constructed by WGCNA, in which each node represents a gene, and the connecting line (edge) between genes represents the co‐expression correlation. The genes with edge weights >0.1 are visualized by Cytoscape. The size and colour of each circle represent the number of edges.

The module MElightcyan contained 46 genes, including 29 transcription factors. The expression pattern of these genes coincided with the stage of flower bud differentiation, which was expressed at 3TLS, and the expression levels of these genes in the two early‐maturing varieties were always higher than those in the two late‐maturing varieties (Figure 4a). Hub genes were those that showed the most connections in the network, including *CAL* homologous genes (*GH_D07G0876*), *AP1* homologous genes (*GH_A04G1749*), *AGL6* homologous genes (*GH_D09G0468*), *GhMADS22*, *GHMADS23* and *GhSOC1* (Figure 4c and Table S4). Among these, *GhMADS23*, *GhMADS22* and *GhSOC1* have been proven to be involved in the regulation of cotton flowering, and their overexpressions in *Arabidopsis* could significantly advance flowering time (Su *et al*., 2016; Zhang *et al*., 2013; Zhang *et al*., 2016). In this study, these three genes showed lower expression from 0TLS to 2TLS in both early‐ and late‐maturing varieties. However, their expression levels in early‐maturing varieties from 3TLS were significantly higher than those in two late‐maturing varieties, which coincided with previous results (Figure S2). The above results further showed the reliability of the data. The genes in the module MElightcyan might play an important role in regulating the transition from vegetative to reproductive growth in cotton. Most notably, *GH_D07G0876* had the largest number of connecting lines (edges), which was the homologous gene of *AtCAL* gene, named *GhCAL* (Figure 4c and Table S4). Overexpression of *Arabidopsis AtCAL* could promote early flowering (Li *et al*., 2000). However, except in *A. thaliana*, the *CAL* gene was rarely reported in other plants.

Interestingly, some genes were only expressed at 5TLS in the two early‐maturing varieties (Figure 4a). These genes were from the ABCDE model. ABCDE genes were the most widely known and well‐studied in flower development (Krizek and Fletcher, 2005). The class A gene had four *AP1* homologous genes, class B included three *PI* homologous genes, and class E had the highest number of genes, that is two *SEP2* homologous genes, four *SEP3* genes and three *SEP4* genes (Figure 4b). These genes were expressed only in floral organs (Figure S3).

### The function of *GhCAL* in regulating transition from vegetative to reproductive growth

The expression level of *GhCAL* in the roots was low, whereas that in the shoot apex was the highest. The expression of *GhCAL* could also be detected in the leaves, stems and buds (Figure S4). To explore the function of *GhCAL* in flowering regulation, three *35S::GhCAL* transgenic *A. thaliana* lines were constructed (Figure S5a). qRT‐PCR analysis with specific primers showed that *GhCAL* expression was significantly higher than that of wild‐type plants (Figure S5b). Under long‐day conditions, the flowering times of *GhCAL* transgenic *A. thaliana* were 3–5 days earlier than that of wild‐type plants (Table S5). The growth of some transgenic plants was bifurcated (Figure S5a), which changed the plant structure of *A. thaliana*. These results suggested that overexpression of *GhCAL* could promote the transition of *A. thaliana* from vegetative to reproductive growth.

**Figure 5:**
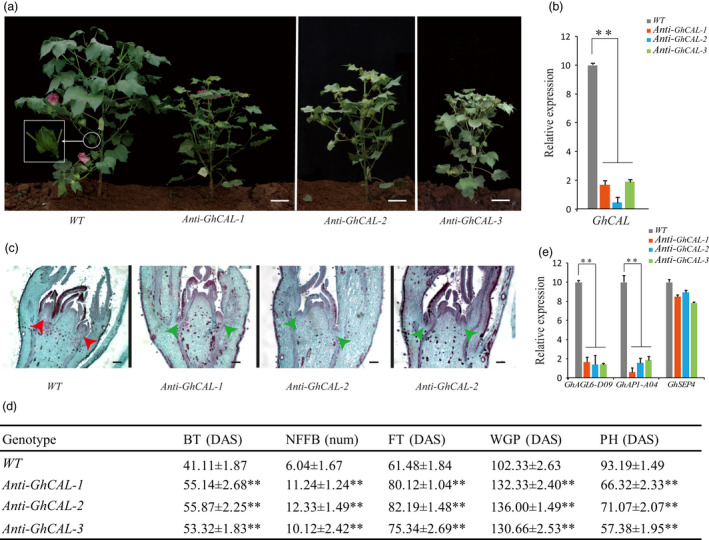
Phenotype of transgenic cotton lines with *Anti‐GhCAL*. (a) Morphological comparison of ZM24 (*WT*) and transgenic cotton lines with *Anti‐GhCAL*. The white box is the white circle area magnified three times. Scale bars, 8 cm. (b) Relative transcript level of *GhCAL* in *WT* and T_3_ transgenic cotton lines. (c) Paraffin section of ZM24 (*WT*) and transgenic cotton lines with *Anti‐GhCAL* shoot apexes at 3TLS. Scale bars, 135 μm. (d) Comparison of characters related to early maturity between transgenic and wild‐type cotton. Plants sown in the Experimental Field of Cotton Research Institute, Chinese Academy of Agricultural Sciences, Anyang, Henan Province (36°08′N, 114°48′E). BT, budding time; DAS, days after sowing, shown as the average ± standard deviation (SD); FT, flowering timing; NFFB, node of the first fruiting branch; PH, plant height; WGP, whole growth period. (e) Relative transcript levels of *GhAGL6‐D09*, *GhAP1‐A04* and *GhSEP4* in *WT* and T_3_ transgenic cotton lines. **Significantly different from wild type at *P* < 0.01, error bars are standard deviations of three biological replicates.

To further confirm the functional role of *GhCAL* in cotton, an antisense expression vector containing the antisense sequence full‐length coding region of *GhCAL* driven by a 35s promoter was constructed and transformed into ZM24 (an early‐maturing cotton variety). A total of seven T_3_ transgenic cotton lines were obtained (Figure S6a). Among them, the expression of *GhCAL* in five transgenic lines decreased significantly (Figure S6b). Three T_3_ transgenic cotton lines with *GhCAL* silencing were further studied, namely *Anti‐GhCAL‐1*, *Anti‐GhCAL‐2* and *Anti‐GhCAL‐3* (Figure 5a). qRT‐PCR analysis with specific primers confirmed that the transcript level of *GhCAL* was significantly lower than that in wild‐type plants (Figure 5b). Flower bud differentiation in *Anti‐GhCAL* transgenic cotton plants occurred later (Figure 5c). Compared with the wild type, the budding times of the three T_3_ transgenic lines were delayed by 14, 15 and 12 days, and the flowering times were delayed by 19, 21 and 14 days, respectively. The first fruit branches of wild‐type plants usually occurred in the sixth or seventh node of the main stem, while in transgenic cotton, they occurred in the 11th node, 12th node and 10th node, respectively (Figure 5d). In terms of plant height, the three transgenic cotton lines were significantly shorter than wild‐type plants. The above results showed that the decrease in *GhCAL* expression delayed the transition from vegetative to reproductive growth in cotton.

### Virus‐induced silencing of *GhAGL6‐D09* and *GhAP1‐A04* delayed flowering in cotton

To explore how *GhCAL* regulated cotton flowering, the expressions of other genes with more connecting lines (edges) in the *GhCAL*‐centric WGCNA gene network were analysed (Figure 4c). *GH_D09G0468*, which encoded the homologous gene of *AGL6*, and *GH_A04G1749*, which encoded the homologous gene of *AP1*, were designated as *GhAGL6‐D09* and *GhAP1‐A04*, respectively. *GhAGL6‐D09* was expressed in the leaves, shoot apices, flower buds and stems. There was lowest expression of *GhAP1‐A04* in the shoot apices, whereas the highest expression was observed in the flower buds (Figure S4). In *Anti‐GhCAL* transgenic cotton lines, the expressions of *GhAGL6‐D09* and *GhAP1‐A04* were significantly lower, which might be due to the decrease in *GhCAL* expression (Figure 5e). In the *35S::GhCAL* transgenic *A. thaliana* lines, *GhCAL* was significantly up‐regulated compared to the wild‐type plants, whereas *AGL6* and *AP1* were also significantly up‐regulated, by nearly 220‐ and 2300‐fold, respectively (Figure S5b). These results suggested that *AGL6* and *AP1* were regulated by *GhCAL* and acted as important regulatory factors in controlling the transition from vegetative to reproductive growth.

To further verify the above results, we constructed *35s::GhAGL6‐D09* and *35s::GhAP1‐A04* overexpression vectors and transformed *A. thaliana*. Three T_3_ transgenic *A. thaliana* lines were obtained. Compared with wild type, the flowering times of *35s::GhAGL6‐D09* and *35s::GhAP1‐A04* transgenic *A. thaliana* were significantly earlier, and the numbers of rosette leaves decreased (Figure S7a,c and Tables S6 and S7). In *35s::GhAGL6‐D09* transgenic plants, the expression of *CAL* was slightly up‐regulated. However, the expression of *AP1* increased by a certain multiple, suggesting that *AGL6* might also regulate the expression of *AP1* to some extent (Figure S7b). In *35S::GhAP1‐A04* transgenic plants, the expression of *AGL6* did not change. However, the expression of *CAL* increased by a certain multiple, indicating that *AP1* might have a feedback regulation mechanism for *CAL* (Figure S7d).

**Figure 6:**
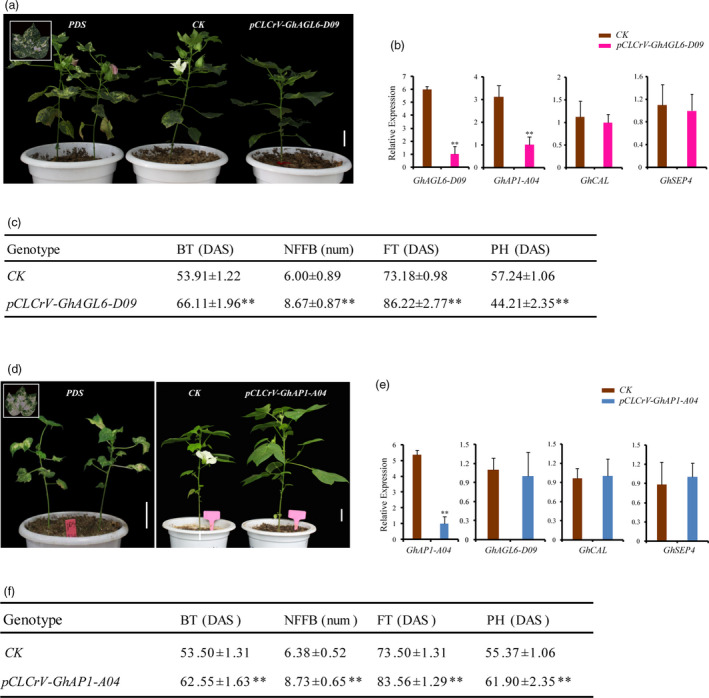
Functional analysis of *GhAL6‐D09* and *GhAP1‐A04* via VIGS. (a) VIGS of *GhAGL6‐D09* in cultivar ZM24. ZM24 with *pCLCrVA::00* was used as control (*CK*). *PDS*: *pCLCrVA::PDS*. Scale bars, 4 cm. (b) Relative transcript levels of *GhAGL6‐D09*, *GhCAL*, *GhAP1‐A04* and *GhSEP4* in *CK* and VIGS plants. *CK* and *pCLCrV‐GhAGL6‐D09* shown as the average ± standard deviation. (c) Phenotype of virus‐induced gene silencing plants of *GhAGL9‐D09*. (d) VIGS of *GhAP1‐A04* in cultivar ZM24. ZM24 with *pCLCrVA::00* was used as *CK*. *PDS*: *pCLCrVA::PDS*. Scale bars, 4 cm. (e) Relative transcript levels of *GhAP1‐A04*, *GhCAL*, *GhAGL6‐D09* and *GhSEP4* in *CK* and VIGS plants. (f) Phenotype of virus‐induced gene silencing plants of *GhAP1‐A04*. *CK* and *pCLCrV‐GhAGL6‐D09* shown as the average ± standard deviation. ** Significantly different from wild type at *P* < 0.01.

The functions of *GhAGL6‐D09* and *GhAP1‐A04* were further verified by virus‐induced gene silencing (VIGS) in cotton. qRT‐PCR showed that VIGS plants had high gene silencing efficiency, and the expressions of *GhAGL6‐D09* and *GhAP‐A04* significantly decreased in their corresponding silenced plants (Figure 6b,e). Compared with non‐VIGS (*CK*) plants, the budding and flowering times of silenced plants were significantly later, and the nodes of the first fruiting branch were higher (Figure 6a,c,d,f). Interestingly, the *GhAGL6‐D09*‐silenced plants were shorter, which was consistent with the phenotypic changes in the *Anti‐GhCAL* transgenic cotton lines, and the expression of *GhAP1‐A04* significantly decreased, whereas the expression of *GhCAL* was stable (Figure 6b). However, the *GhAP1‐A04*‐silenced plants were taller, and the expression levels of *GhAGL6‐D09* and *GhCAL* did not significantly differ from those of the *CK* (Figure 6e). The above results showed that *GhAGL6‐D09* and *GhAP1‐A04* played an important role in regulating flowering in cotton, and *GhAGL6‐D09* regulated the expression of *GhAP1‐A04*.

### *GhCAL‐D07* could form heterodimers with *GhAGL6‐D09/GhAP1‐A04*

**Figure 7:**
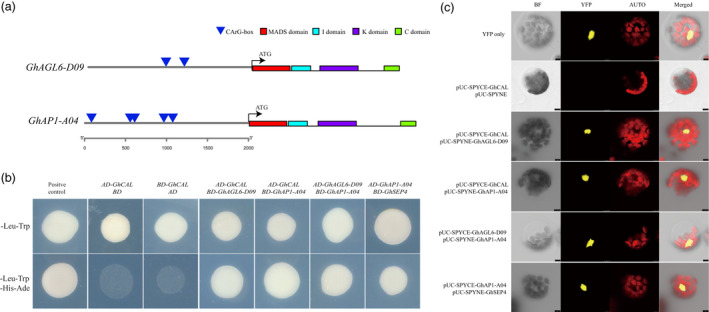
Protein interaction of *GhCAL*, *GhAP1‐A04* and *GhAGL6‐D09*. (a) Prediction of *GhAGL6‐D09* and *GhAP1‐A04* gene domains and CArG‐box in 2K upstream region of their transcriptional start site. (b) Yeast two‐hybrid assay for protein interactions. Cell growth on ‐Leu‐Trp dropout selective medium represents normal cells (upper panel), and ‐Leu‐Trp‐His‐Ade dropout selective medium represents positive interactions (bottom panel). (c) Bimolecular fluorescence complementation (BiFC) assay of protein interactions of GhCAL, *GhAGL6*‐D09, GhAP1‐A04 and GhSEP4 in *Arabidopsis* protoplasts. AUTO, autofluorescence; BF, brightfield; Merged, merge of YFP, AUTO and BF; YFP, YFP fluorescence. Bars, 4 μm.

*GhCAL*, *GhAGL6‐D09* and *GhAP1‐A04* belong to the MADS transcription factor family and have four characteristic domains, namely MADS, I, K and C, and there were five CArG‐box elements in *GhAP1‐A04* and two CArG‐box elements in *GhAGL6‐D09* (Figure 7a). Previous studies have shown that some MADS‐box transcription factors may form heterodimers to play a regulatory role (Shore and Sharrocks, 1995). To confirm interactions among GhCAL, GhAGL6‐D09 and GhAP1‐A04, yeast two‐hybrid (Y2H) and bimolecular fluorescence complementary (BiFC) detection were conducted. Y2H analysis showed that the three proteins interacted with each other, forming heterodimers (Figure 7b). These interactions were further verified in a BiFC test using *Arabidopsis* protoplasts (Figure 7c). GhCAL formed heterodimers with GhAGL6‐D09 and GhAP1‐A04. At the same time, *GhCAL* regulated the expressions of *GhAGL6‐D09* and *GhAP1‐A04*. In *A. thaliana*, MADS‐box transcription factors could form specific heterodimers that bound to the CArG‐box in the promoter region of themselves and regulate their own expression (Shore and Sharrocks, 1995; Trobner *et al*., 1992). GhCAL might form heterodimers with GhAGL6‐D09 and GhAP1‐A04 by binding to the CArG‐boxes in their promoter regions and then regulated their expressions.

## Discussion

In China, with the decrease in cultivated land area, the competition for land between grain and cotton has become severe. There are many weather disasters in spring in the cotton areas of northern Xinjiang, and a certain area is replanted every year due to these disasters, resulting in late sowing date and high risk of cotton planting, so there is an urgent need for early‐maturing cotton varieties in production (Li *et al*., 2002a). Although there were many studies on the early maturity of cotton, current understanding of its molecular regulatory mechanism network is limited. This study has shown that flower bud differentiation in cotton occurs in the third true leaf stage (3TLS). To date, no transcriptomic study of shoot apices in cotton at the seedling stage has been conducted. Here, we selected two early‐maturing and two late‐maturing varieties, collected shoot apex samples before and after flower bud differentiation and analysed their transcript dynamics. Finally, we identified a key regulatory gene that regulated the transition of cotton from vegetative to reproductive growth. The gene was transformed into cotton for functional verification and regulatory interaction network analysis. Our findings will be helpful to future functional research studies on the regulation of upland cotton flowering network. The transgenic plants developed in this study can also be used for breeding of early‐maturing cotton varieties. More broadly, our results provide a better understanding of the complexity of upland cotton and can guide future experimental studies.

### High‐resolution temporal dynamic transcriptome landscape of the transition from vegetative to reproductive growth of early‐ and late‐maturing cotton varieties

In cotton development, the transition from vegetative to reproductive growth is essential to the early maturity of cotton. Flower bud differentiation occurred at 3TLS in early‐maturing varieties and 5TLS in late‐maturing varieties (Figure 1). To establish its gene regulatory network, we generated a comprehensive high‐resolution temporal dynamic transcriptome landscape using 72 cotton shoot apex samples between two early‐maturing and late‐maturing cotton varieties. The samples of this study completely covered the complete seedling development stage of two late‐maturing varieties and two late‐maturing varieties from 0TLS to 5TLS, including vegetative and reproductive growth stages. This is the first time to conduct a comprehensive large‐scale sequencing of the part of flower bud differentiation, that is the shoot apexes of cotton. The analysis of dynamic transcriptome data clearly showed each developmental stage and the differences between early‐ and late‐maturing varieties. These huge transcriptome data provide rich resources for future functional research, which will greatly improve our understanding of the genetic control of cotton growth phase transition.

### Candidate genes in module MElightcyan regulating the transition from vegetative to reproductive growth in cotton

Flower bud differentiation is a symbol of transition of plants from vegetative to reproductive growth. It is not only a process of morphogenesis, but also an extremely complex biological process that is regulated by a large number of genes, forming a complex regulatory network. Using pairwise comparison and weighted gene co‐expression network analysis, we identified 10 modules of co‐expressed genes (Figure 3a,b). The gene expression pattern in module MElightcyan coincided with the initial stage of flower bud differentiation. This module contains 46 genes, nine of which have been cloned from upland cotton and have been confirmed to be associated with flowering or floral development, including *GhMADS23*, *GhMADS22*, *GhSOC1*, *GhMADS42*, *GhMADS13*, *GhMADS11* and *GhMADS1* (Jiang *et al*., 2013; Zheng *et al*., 2004; Zhang *et al*., 2013; Zhang *et al*., 2016). These genes were also differentially expressed at 3TLS, further confirming the accuracy of the results of our histological and transcriptome analyses. Three new genes, namely *GhCAL*, *GhAGL6‐D09* and *GhAP‐A04*, which encoded MADS transcription factors, were found in this module. In *A. thaliana*, the overexpression of the *AtCAL* gene promoted early flowering (Li *et al*., 2000). *AtAP1* has been shown to promote flowering (Bowman *et al*., 1993; Weigel and Nilsson, 1995). *AtAGL6* induced early flowering by limiting the expression of flowering inhibitors *FLC* and *MAF* or by promoting the expression of circadian clock factor *ZTL* (Koo *et al*., 2010; Yoo *et al*., 2011). Overexpression of *OMADS1*, an Orchid gene closest to the *AGL6* sequence, led to early flowering and loss of inflorescence uncertainty in *A. thaliana* (Hsu *et al*., 2003). In summary, the genes in module MElightcyan might play an important role in flower bud differentiation. The identified novel genes provide information on the regulatory pathway of cotton flower bud differentiation as well as genetic resources for the development of early‐maturing cotton varieties.

### *GhCAL* mediates the regulatory pathway from vegetative to reproductive growth by regulating *GhAP1‐A04* and *GhAGL6‐D09*

WGCNA showed that *GhCAL*, which encoded a MADS transcription factor, has the most connections within the network, suggesting that it played essential roles. To verify the function of *GhCAL*, *35S::GhCAL* and *35S::GhCAL‐antisense* were transformed into cotton, and *35S::GhCAL* was transformed into *A. thaliana*. Unfortunately, none T_0_ transgenic cotton of overexpressing *GhCAL* could bear bolls, and the underlying mechanism remains unclear. *GhCAL* silencing in transgenic cotton plants resulted in late flowering within 14–19 days. Overexpression of *GhCAL* in *A. thaliana* induced early flowering and plant type change. Interestingly, the expression of *GhAGL6‐D09* and *GhAP1‐A04* significantly decreased in *Anti‐GhCAL* transgenic cotton plants, which suggested that *GhAGL6‐D09* and *GhAP1‐A04* were directly regulated by *GhCAL*. The ectopic expressions of *GhAGL6‐D09* and *GhAP1‐A04* in *A. thaliana* significantly advanced the flowering times and reduced the numbers of rosette leaves. *GhAGL6‐D09*‐ and *GhAP1‐A04*‐silenced cotton plants clearly showed late flowering. In *GhAGL6‐D09*‐silenced cotton plants, the expression of *GhAP1‐A04* was significantly reduced, which suggested that *GhAGL6‐D09* regulated the expression of *GhAP1‐A04*. The above results showed that *GhAGL6‐D09* and *GhAP1‐A04* also played important roles in regulating stage transition in cotton.

GhCAL, GhAGL6‐D09 and GhAP‐A04 were members of the MADS family and possessed the MADS, I, K and C domains. Interactions between MADS‐box proteins were necessary to properly perform their functions (Theissen, 2001). Our results showed that GhCAL could form heterodimers with GhAGL6‐D09 and GhAP1‐A04, and GhAGL6‐D09 forms heterodimers with GhAP1‐A04. Plant MADS proteins, as dimers, bound to their common DNA‐binding site, CArG‐box (Schwarz‐Sommer *et al*., 1992; Shore and Sharrocks, 1995). The heterodimers of DEFA and GLO combined with the CArG‐box sequence in the promoter regions of their own genes to establish product self‐regulation and control (Trobner *et al*., 1992). *PI* and *AP3* formed heterodimers, which bound to three CArG‐boxes of the *AP3* promoter and regulated its expression (Samach *et al*., 1997). Five CArG‐boxes in the *GhAP1‐A04* promoter region and two CArG‐boxes in the *GhAGL6‐D09* promoter region were identified. These results suggested that a similar regulatory mechanism might exist in cotton. The heterodimers formed by GhCAL, GhAGL6‐D09 and GhAP1‐A04 might bind to the CArG‐boxes in the promoter regions of GhAGL6‐D09 and GhAP1‐A04 and then regulated the expressions of these two genes. However, the existence of this regulatory model needs to be verified by further experiments. Based on the above results, we confirmed that *GhCAL* regulated the transition from vegetative to reproductive growth of cotton by regulating *GhAGL6‐D09* and *GhAP1‐A04*.

Surprisingly, contrary to previous understanding, the height of *Anti‐GhCAL* transgenic cotton plants decreased. *GhAGL6‐D09*‐silenced plants also showed a decrease in height, whereas *GhAP1‐A04*‐silenced plants were taller. In rice, both *OsMADS1* and *OsMADS32* mutations led to a significant decrease in plant height (Feng *et al*., 2013; Wang *et al*., 2017), suggesting that MADS genes imparted multiple regulatory effects. The expression of *GhAGL6‐D09* was highest in stems (Figure S4). In *Anti‐GhCAL* transgenic plants, the expression of *GhAGL6‐D09* significantly decreased. Based on the above results, we hypothesized that the change in height in *Anti‐GhCAL* transgenic plants was caused by a decrease in *GhAGL6‐D09* expression. In cotton, *GhAGL6‐D09* regulated not only flowering but also plant height. However, this result requires further verification.

### The interaction between *GhAP1‐A04* and *GhSEP4* regulates sepal development

The ABCDE classes of genes are the most well‐known and extensively studied flower development‐related genes (Krizek and Fletcher, 2005). There were five rounds of floral structural development in floral organs. The sepal was controlled by the A and E genes, the petals were controlled by the A, B and E genes, the stamens were controlled by the B, C and E genes, the carpel was controlled by the C and E genes, and ovules were controlled by the C, D and E genes (Ditta *et al*., 2004; Krizek and Fletcher, 2005). Studies had shown that cotton had the same conservative flower bud differentiation and development process as *A. thaliana* (Shen *et al*., 1989). There were four *AP1* homologous genes in class A genes, three homologous genes of *PI* gene in class B genes, and two *SEP2* homologues, four *SEP3* genes and three *SEP4* genes in class E genes in module MElightcyan (Figure 4b). These genes are specifically expressed in cotton floral organs (Figure S3). It had been proven that AP1 and SEP3 interacted with each other to control sepal development (Fan *et al*., 1997; Pelaz *et al*., 2001). Interestingly, in cotton, the *AP1* and *SEP4* genes are highly expressed in sepals, whereas *SEP3* gene expression is very low. This indicated that there were differences in *SEP* members that controlled sepal development in cotton. Yeast two‐hybrid and BiFC experiments also proved that GhAP1‐A04 could form a heterodimer with GhSEP4 (Figure 7b,c). However, how these two genes control sepal development requires further verification. In *A. thaliana*, AP1, SEP3, PI and AP3 genes formed protein tetramers that controlled petal development (Ditta *et al*., 2004; Krizek and Fletcher, 2005), but it is noteworthy that the *AP3* gene has not been detected in this study. The results showed that in early‐maturing cultivars, after flower bud differentiation in 3TLS, the flower organs only began sepal differentiation at 5TLS. Flower development in cotton might have a conservative ABCDE model. Interestingly, we found that there was no significant change in the expression of *GhSEP4* in transgenic plants (Figures 5e and 6b,e). This suggested that *GhSEP4* might exist in other regulatory pathways. Overexpression of *GhAP1‐A04* did not cause changes in floral organs, which was due to the lack of expression of *GhSEP4*. In summary, in cotton, early‐maturing cultivars had sepal development at 5TLS, which was regulated by the interaction between *GhAP1‐A04* and *GhSEP4*.

**Figure 8:**
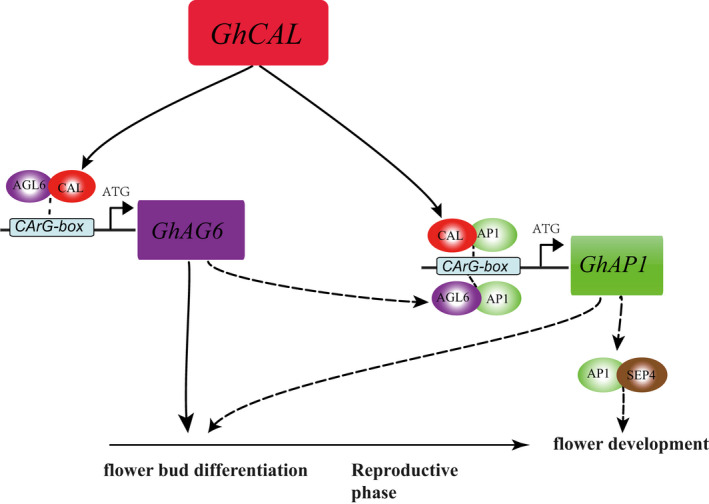
*GhCAL*‐mediated regulatory pathway for the transition from vegetative to reproductive growth. Squares represent nucleic acids, and ovals indicate proteins. Solid arrows represent the regulatory relationship that was verified in this study, and the dashed arrows indicate the predicted regulatory relationship.

Finally, the Hub gene in the module MElightcyan, *GhCAL* was proven to regulate the expression of *GhAGL6‐D09* and *GhAP1‐A04* and promoted the transition of cotton from vegetative to reproductive growth. We proposed a working model for flowering induction by *GhCAL* (Figure 8). In early‐maturing varieties, *GhCAL* was expressed at 3TLS. GhCAL formed a heterodimer with GhAGL6‐D09, which might bind to the CArG‐box of the *GhAGL6‐D09* promoter region and induce the expression of *GhAGL6‐D09* to initiate flower bud differentiation. At 5TLS, GhCAL/GhAGL6 formed a heterodimer with GhAP1‐A04, which might bind to the CArG‐box of the *GhAP1‐A04* promoter region and induce the expression of *GhAP1‐A04*. The *GhAP1‐A04* and *GhSEP4* genes in the ABCDE model regulated the development of cotton floral organs. In late‐maturing varieties, *GhCAL* was expressed at 5TLS, which led to the late initiation of flower bud differentiation. In general, *GhCAL* acted as the regulatory centre of the entire pathway, regulating the transition from vegetative to reproductive phase to induce early flowering.

## Experimental procedures

### Plant materials and sample collection

The early‐maturing upland cotton varieties CCRI50 and Yanzao2 and late‐maturing varieties Guoxinmian11 and STS458 were planted at the Cotton Research Institute of the Chinese Academy of Agricultural Sciences Experimental Field in Anyang City, Henan Province, China, and managed according to general field management. Shoot apexes at six stages were collected: the cotyledon stage (0TLS), the first true leaf stage (1TLS), the second true leaf stage (2TLS), the third true leaf stage (3TLS), the fourth true leaf stage (4TLS) and the fifth true leaf stage (5TLS). For each stage, 10–15 shoot apexes of the cotton seedlings were pooled together for each of the three biological replicates for RNA extraction. These samples were immediately placed into liquid nitrogen and stored in a freezer at −80 °C.

### Anatomical observation on shoot apexes

In this study, three to five shoot apexes of the cotton seedlings were gently excised to keep samples intact for each of the three biological replicates at each stage. The plant samples were immersed in FAA stationary solution (70% ethanol : formaldehyde : acetic acid, 9 : 1 : 1), and the fixed samples were dehydrated, embedded in the accessory membrane and sectioned. Finally, fan red dye was used for staining (Cai *et al*., 2018). The process of cotton flower bud differentiation was observed under an optical microscope (Olympus SZ61TR, Olympus, Tokyo, Japan), and the differentiation date and morphological characteristics of each period were recorded and photographed.

### RNA isolation and sequencing

Total RNA was extracted with TRIzol reagent (Invitrogen, California, USA). According to the manufacturer's recommendation, the NEBNext Ultra RNA Library Prep Kit for Illumina (New England Biological system, Massachusetts, USA) was used to generate a digital gene expression library. RNA‐seq libraries were sequenced to generate 150‐nucleotide paired‐end reads on an Illumina HiSeq platform. These sequence data have been submitted to the NCBI databases under accession number PRJNA598731.

### Reads mapping and analysis

The TM‐1 reference genome (*Gossypium hirsutum*, ZJU) (Hu *et al*., 2019) was downloaded from https://cottonfgd.org/about/download.html. After removing low‐quality reads, Illumina sequencing reads were mapped to the TM‐1 reference genome using TopHat (Trapnell *et al*., 2009) with default settings for parameters. FPKM (Trapnell *et al*., 2009) was used to quantitatively estimate the value of gene expression. DESeq was used to analyse the differential expression of genes, and the genes with |log_2_ratio ≥ 1 and *q* < 0.05 were selected for follow‐up analysis. Each stage in the same variety was compared with the previous stage, and the early‐maturing varieties were compared with the same stage of the late‐maturing varieties, respectively. For the precise identification of differentially expressed genes between early‐ and late‐maturing varieties, the differential genes between two early‐maturing varieties and between two late‐maturing varieties at the same stage were excluded from the analysis.

The relative FPKM value (FPKM^geneX in variety_stageX^/Average FPKM^geneX across all variety_stages^) of differentially expressed genes was calculated to identify genes with low absolute FPKM value (such as transcription factors) but significant changes in specific variety or stages. The result was designated as NFPKM.

### WGCNA and functional enrichment analysis

Co‐expression networks were built using WGCNA in BMKCloud (www.biocloud.net). A total of 5312 genes were used for WGCNA, and the average FPKM was introduced into WGCNA. These modules were obtained using the automatic network build function block with default settings. The eigenvalues of each module were calculated and used to test the association with each variety and stage. These genes were grouped into 10 variety‐ and stage‐specific modules. The networks were visualized using Cytoscape_v.3.0.0 (Otasek *et al*., 2019).

AgriGO (Tian *et al*., 2017) was used for GO analysis. The correct error detection rate of GO terminology is *0.05*, which is considered to be significantly enriched.

### Cloning, vector construction and transformation of candidate genes

Using the shoot apex cDNA of early‐maturing variety CCRI50 as template, the complete CDS sequence of the candidate genes was amplified by PimerSTAR GXL DNA Polymerase (Takara, Shiga, Japan). The amplification conditions were as follows: 98 °C for 1 min; followed by 30 cycles of 98 °C at 10 s, 55 °C for 15 s and 68 °C for 2 min. The amplified product was cloned into vector PBI121. The antisense sequence of *GhCAL* (*GhCAL‐antisense*) was also constructed into the overexpression vector PBI121. *A. thaliana* plants were transformed by *Agrobacterium tumefaciens*‐mediated gene transfer as described elsewhere (Clough and Bent, 1998). Through DNA transfer mediated by *Agrobacterium* and a series of co‐culture including callus, differentiated callus, embryogenic callus and grafted plant, the *GhCAL‐antisense* fusion gene was transformed into cotton ‘ZM24’ hypocotyl (Li *et al*., 2002b). The primers used in this study are shown in Table S8.

### Sequence alignment and phylogenetic analysis

In this study, nucleic acid and protein sequences were downloaded from CottonFGD (https://cottonfgd.org/). ClustalW (http://www.ebi.ac.uk) was used for multiple sequence alignment. The phylogenetic tree was constructed by adjacency method in MEGA5.05 (Tamura *et al*., 2007), a software of molecular evolutionary genetics analysis. The reliability of the nodes in the tree was evaluated by 1000 repeated bootstraps.

### Protein interaction in Y2H assays

The coding regions of candidate genes were amplified by specific primers PCR and cloned into a pGBKT7 vector, and then, the self‐activation activity assays and toxicity tests were conducted. The coding region of the candidate genes was cloned into a pGADT7 vector. PGBKT7‐gene and pGADT7 were used as negative control plasmids. The recombinant plasmids were introduced into yeast strain Y2Hs. The two‐hybrid interaction was detected on selective SD/‐Trp/‐Leu double shedding and SD/‐Ade/‐His/‐Leu/‐Trp quadruple medium.

### BiFC

Open reading frames (ORFs) of full‐length genes were inserted into separate pUC‐SPYNE and pUC‐SPYCE vectors. BiFC measurement was conducted according to the previous scheme (Schutze *et al*., 2009; Walter *et al*., 2004). Protoplasts isolated from *Arabidopsis* leaves were used for plasmid transformation. Confocal microscopy was performed using FV1000 instruments (Olympus, Tokyo, Japan).

## Conflict of interest

The authors declare that they have no competing interests.

## Authors' contribution

S. Yu, H. Wei and H. Wang designed the experiments. P. Chen performed the WGCNA. Z. Su and P. Hao conducted cloning, vector construction and transformation of candidate genes. L. Ma, J. Zhang, Q. Ma, J. Liu and G. Liu performed field cultivation of cotton plants and sample collection. S. Cheng and Z. Su performed the yeast two‐hybrid assay. S. Cheng analysed the results and wrote the manuscript. S. Yu, H. Wang and H. Wei revised the manuscript. All of the authors have reviewed and approved the final manuscript.

## Supporting information


**Figure S1** Pearson correlation between samples. The colors of the boxes represent the degree of correlation; red represents the highest degree of correlation and blue indicates the lowest degree of correlation.

**Figure S2** FPKM of *GhMADS22*, *GhMDS23*, and *GhSOC1* in different developmental stages of four varieties.

**Figure S3** Sixteen ABCDE genes from the module MElightcyan were expressed in different tissues of cotton, and the data were from CottonFGD (Zhu *et al*., 2017).

**Figure S4** Relative expressions of *GhCAL*, *GhAGL6‐D09*, and *GhAP1‐A04* in different tissues of *G. hirsutum*. Error bars are standard deviations of three biological replicates.

**Figure S5** Phenotype of *Arabidopsis thaliana* with *GhCAL* overexpression. (a) Morphological comparison of *GhCAL* overexpression transgenic *A. thaliana* lines and the wild type (*WT*). Scale bars, 4 cm. (b) Relative transcript levels of *GhCAL* and other *Arabidopsis* flowering genes in wild type *Arabidopsis* (*WT*) and transgenic *Arabidopsis* lines.

**Figure S6** Overexpression of the antisense sequence full‐length coding region of *GhCAL* in cotton. (a) PCR detection of antisense fragment of *GhCAL* in transgenic cotton. line1: DNA Marker 3; line2: *WT*; line3‐7: *Anti‐GhCAL‐1‐7*. (b) Relative transcript level of *GhCAL* in *WT* and T_3_ transgenic cotton lines. **Significantly different from wild type at *P* < 0.01, error bars are standard deviations of three biological replicates.

**Figure S7** Overexpression of *GhAGL6‐D09* and *GhAP1‐A04* in *Arabidopsis thaliana* promotes flowering. (a) Morphological comparison of *GhAGL6‐D09* overexpression transgenic *A. thaliana* lines and wild type *A. thaliana* (*WT*). Scale bars, 1 cm. (b) Relative transcript levels of *GhAGL6‐D09* and other *Arabidopsis* flowering genes in *WT* and transgenic *Arabidopsis* lines. (c) Morphological comparison of *GhAP1‐A04* overexpression transgenic *A. thaliana* lines and the *WT*. Scale bars, 1 cm. (d) Relative transcript levels of *GhAP1‐A04* and other *Arabidopsis* flowering genes in *WT* and transgenic *Arabidopsis* lines. **Significantly different from *WT* at *P* < 0.01, error bars are standard deviations of three biological replicates.

**Table S1** Summary of RNA‐Seq reads mapping results.

**Table S2** Data of WGCNA analysis.

**Table S3** Enriched GO terms of genes in the 10 modules.

**Table S4** MElightcyan network analysis.

**Table S5** Flowering time‐related phenotypes of *GhCAL* overexpression and wild‐type *Arabidopsis* plants.

**Table S6** Flowering time‐related phenotypes of *GhAGL6‐D09* overexpression and wild‐type *Arabidopsis* plants.

**Table S7** Flowering time‐related phenotypes of *GhAP1‐A04* overexpression and wild‐type *Arabidopsis* plants.

**Table S8** List of all primers used in this study.
