# Supplementary figures and images for: High‐resolution temporal dynamic transcriptome landscape reveals a GhCAL‐mediated flowering regulatory pathway in cotton (Gossypium hirsutum L.)
Source: Plant Biotechnol J. 2020 Aug 3;19(1):153–66. doi: 10.1111/pbi.13449 (PMC7769237; doi:10.1111/pbi.13449)

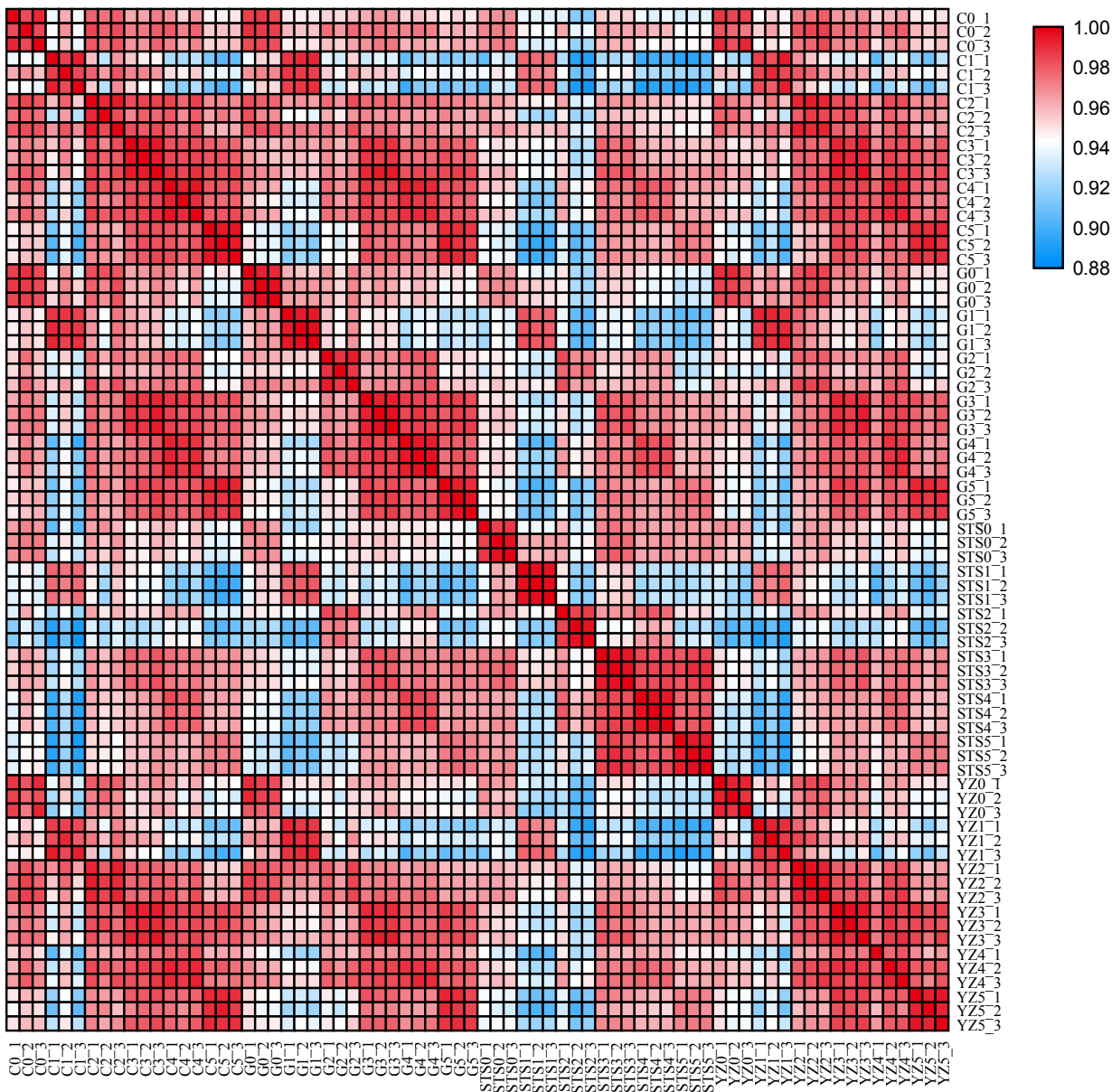

Supplement: Supplementary file 1 — Figure S1 Pearson correlation between samples. The colors of the boxes represent the degree of correlation; red represents the highest degree of correlation and blue indicates the lowest degree of correlation. [file PBI-19-153-s012.pdf]

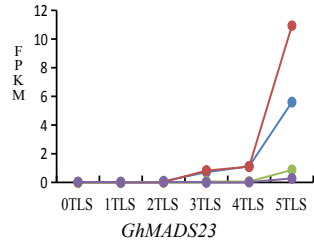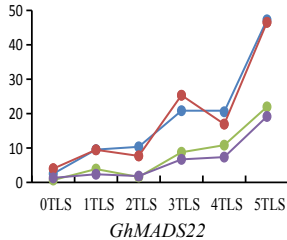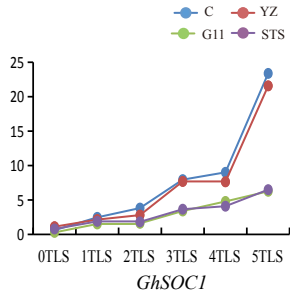

Supplement: Supplementary file 2 — Figure S2 FPKM of GhMADS22, GhMDS23, and GhSOC1 in different developmental stages of four varieties. [file PBI-19-153-s011.pdf]

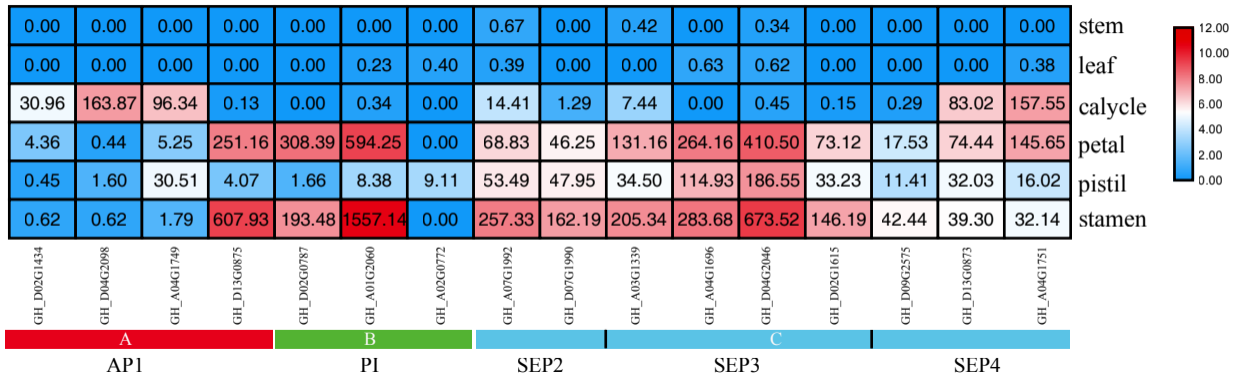

Supplement: Supplementary file 3 — Figure S3 Sixteen ABCDE genes from the module MElightcyan were expressed in different tissues of cotton, and the data were from CottonFGD (Zhu et al., 2017). [file PBI-19-153-s010.pdf]

*GhCAL*

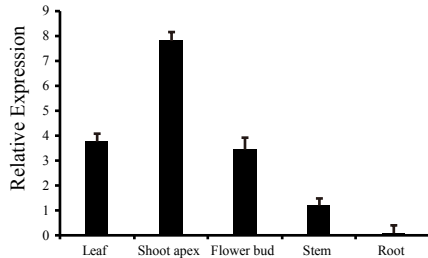

*GhAGL6-D09*

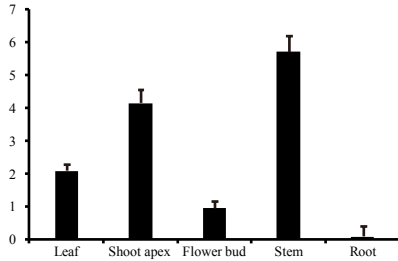

*GhAP1-A04*

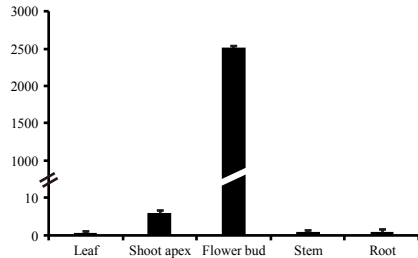

Supplement: Supplementary file 4 — Figure S4 Relative expressions of GhCAL, GhAGL6‐D09, and GhAP1‐A04 in different tissues of G. hirsutum. Error bars are standard deviations of three biological replicates. [file PBI-19-153-s014.pdf]

(a)

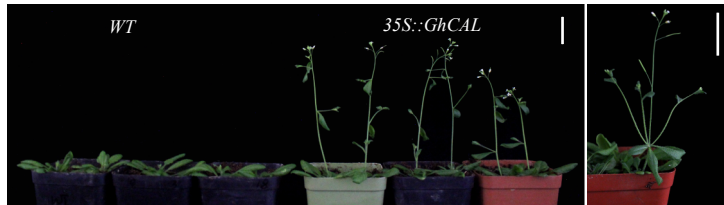

(b)

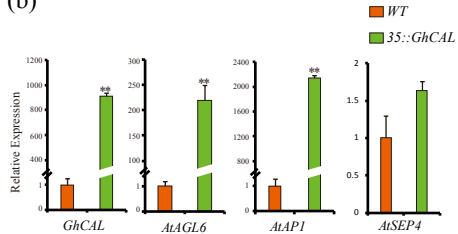

Supplement: Supplementary file 5 — Figure S5 Phenotype of Arabidopsis thaliana with GhCAL overexpression. (a) Morphological comparison of GhCAL overexpression transgenic A. thaliana lines and the wild type (WT). Scale bars, 4 cm. (b) Relative transcript levels of GhCAL and other Arabidopsis flowering genes in wild type Arabidopsis (WT) and transgenic Arabidopsis lines. [file PBI-19-153-s015.pdf]

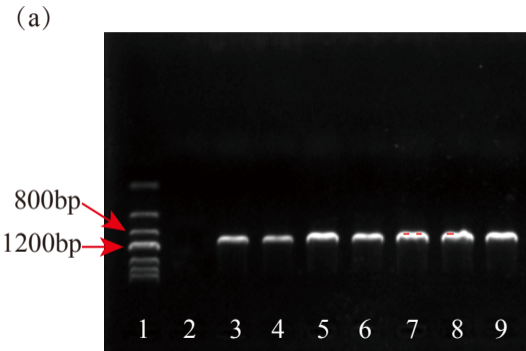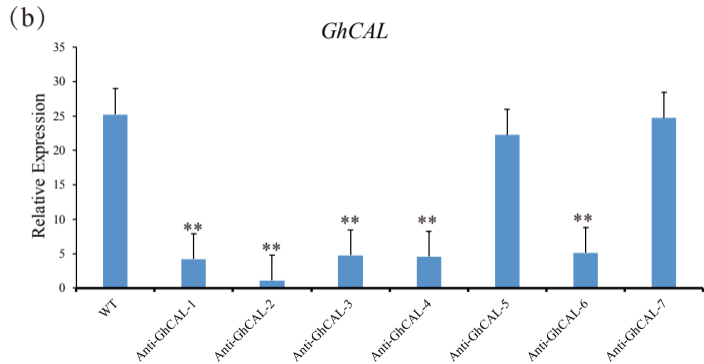

Supplement: Supplementary file 6 — Figure S6 Overexpression of the antisense sequence full‐length coding region of GhCAL in cotton. (a) PCR detection of antisense fragment of GhCAL in transgenic cotton. line1: DNA Marker 3; line2: WT; line3‐7: Anti‐GhCAL‐1‐7. (b) Relative transcript level of GhCAL in WT and T3 transgenic cotton lines. **Significantly different from wild type at P < 0.01, error bars are standard deviations of three biological replicates. [file PBI-19-153-s001.pdf]

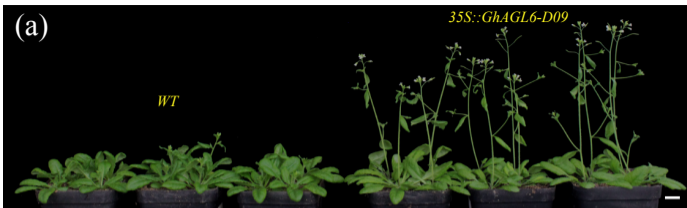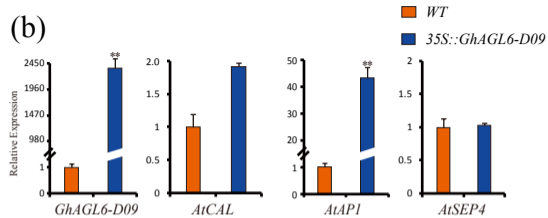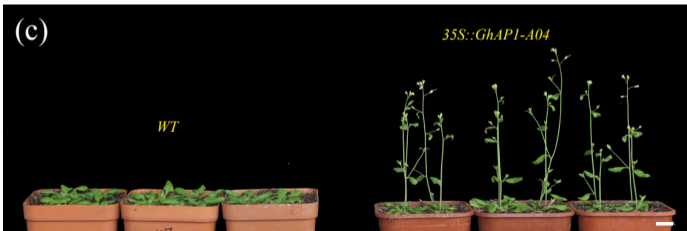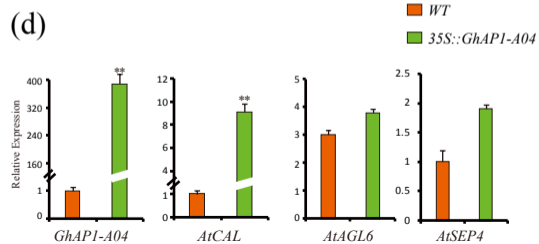

Supplement: Supplementary file 7 — Figure S7 Overexpression of GhAGL6‐D09 and GhAP1‐A04 in Arabidopsis thaliana promotes flowering. (a) Morphological comparison of GhAGL6‐D09 overexpression transgenic A. thaliana lines and wild type A. thaliana (WT). Scale bars, 1 cm. (b) Relative transcript levels of GhAGL6‐D09 and other Arabidopsis flowering genes in WT and transgenic Arabidopsis lines. (c) Morphological comparison of GhAP1‐A04 overexpression transgenic A. thaliana lines and the WT. Scale bars, 1 cm. (d) Relative transcript levels of GhAP1‐A04 and other Arabidopsis flowering genes in WT and transgenic Arabidopsis lines. **Significantly different from WT at P < 0.01, error bars are standard deviations of three biological replicates. [file PBI-19-153-s002.pdf]
